# Electrochemical Selective and Simultaneous Detection of Diclofenac and Ibuprofen in Aqueous Solution Using HKUST-1 Metal-Organic Framework-Carbon Nanofiber Composite Electrode

**DOI:** 10.3390/s16101719

**Published:** 2016-10-17

**Authors:** Sorina Motoc, Florica Manea, Adriana Iacob, Alberto Martinez-Joaristi, Jorge Gascon, Aniela Pop, Joop Schoonman

**Affiliations:** 1 Institute of Chemistry Timisoara of Romanian Academy, Mihai Viteazul 24, Timisoara 300223, Romania; sorinailies@acad-icht.tm.edu.ro; 2 Politehnica University of Timisoara, P-ta Victoriei no.2, Timisoara 300006, Romania; aniela.pop@upt.ro; 3 S.C. DATCOMP S.R.L, Str.Dr.Iosif Nemoianu nr 16/4, Timisoara 300011, Romania; adriana.iacob@datcomp.ro; 4 Materials for Energy Conversion and Storage, Department of Chemical Engineering, Delft University of Technology, Julianalaan 136, Delft 2626 BL, The Netherlands; amjoaristi@yahoo.com (A.M.-J.); j.gascon@tudelft.nl (J.G.); J.Schoonman@tudelft.nl (J.S.)

**Keywords:** electrochemical selective and simultaneous detection, metal-organic framework-carbon nanofiber composite electrode, ibuprofen, diclofenac, cyclic voltammetry, chronoamperometry, multiple-pulsed amperometry

## Abstract

In this study, the detection protocols for the individual, selective, and simultaneous determination of ibuprofen (IBP) and diclofenac (DCF) in aqueous solutions have been developed using HKUST-1 metal-organic framework-carbon nanofiber composite (HKUST-CNF) electrode. The morphological and electrical characterization of modified composite electrode prepared by film casting was studied by scanning electronic microscopy and four-point-probe methods. The electrochemical characterization of the electrode by cyclic voltammetry (CV) was considered the reference basis for the optimization of the operating conditions for chronoamperometry (CA) and multiple-pulsed amperometry (MPA). This electrode exhibited the possibility to selectively detect IBP and DCF by simple switching the detection potential using CA. However, the MPA operated under optimum working conditions of four potential levels selected based on CV shape in relation to the potential value, pulse time, and potential level number, and order allowed the selective/simultaneous detection of IBP and DCF characterized by the enhanced detection performance. For this application, the HKUST-CNF electrode exhibited a good stability and reproducibility of the results was achieved.

## 1. Introduction

The presence of pharmaceuticals in water has been receiving increased attention, taking into account that they belong to the emerging pollutants class. The new and emerging pollutants represent a great challenge due to them not being regulated or currently being in progress of establishing maximum allowance limits [1].

Amongst the main important pharmaceuticals classes that have been reported in environment [2], one of the pharmaceuticals classes that is the most frequently found in water is represented by anti-inflammatories, and diclofenac (DCF) and ibuprofen (IBP) are very representatives for this class. However, studies reporting the toxicity effects on the ecosystem and human health led to including IBP and DCF in the so-called “watch list” of the priority pollutants of the Water Framework Directive [3]. In general, new and emerging pollutants present a new global challenge regarding water quality. Improving water quality worldwide has been recognized as a key for enhanced water safety and security [4]. In this context, there is an urgent need to develop the analytical methods for the quantitative determination of the safety of pharmaceuticals. Various analytical methods have been developed and routinely used for the determination of pharmaceuticals, e.g., chromatography, spectroscopy, and electrochemical methods [5].

Beside the advantages of the electrochemical methods, e.g., high sensitivity, simplicity, good stability, no preparation/preconcentration step requirement, the suitability for the real-time monitoring using low-cost instrumentation represents another very important advantage of the electrochemical methods that favors their use and development.

In the past decades, the voltammetric and amperometric techniques have been used to quantitatively determine pharmaceuticals [6,7,8,9,10]. Taking into account that the key of the efficient electrochemical detection method is represented by the electrode material, various types of solid [11] and carbon-based [12] electrodes have been reported for the determination of pharmaceuticals. The nanomaterials involved in the electroanalysis of pharmaceuticals have been reviewed by A. Rahi et al. [5]. Other types of electrode materials that are specially tailored for desirable properties and that exhibit very good stability are represented by the composite electrodes, whose properties may be a linear combination of the components, or entirely new [13].

To develop the detection protocol for the simultaneous and/or selective determination of pharmaceuticals, the chemically modified composite electrode should be an excellent alternative to avoid interference through a good separation of the detection potentials. Recently, the application of the metal-organic frameworks (MOFs) in electroanalysis—which exhibited electrocatalytic activity towards reduction or oxidation processes of target analytes—has been reported [14,15,16]. Metal–organic framework (MOF), a class of crystalline porous materials with well-defined pore and channel sizes, is composed of repeated metal complex units and can be tailored to meet certain requirements related to a specific application [14]. Several MOF-based electrochemical functional frameworks with good electrochemical properties and electrocatalytic activities have been reported in the electroanalysis [17,18]. MOFs can be directly used as electro-catalysts for fabricating sensors [19]. For example, Zhou et al. [14] have reported very promising results of the Cu-MOF-single-wall carbon nanotubes modified electrode for simultaneous detection of hydroquinone and catechol. One of the most interesting MOFs for practical application is [Cu_3_(BTC)_2_] MOF (BTC = 1,3,5 benzentricarboxilate) that is known as HKUST-1 [20,21]

In this study, the HKUST metal-organic framework-carbon nanofiber composite (HKUST-CNF) electrode has been prepared by film casting method and characterized morphologically and electrically. This electrode was tested to selectively detect ibuprofen (IBP) and/or diclofenac (DCF) simultaneously. To the best of our knowledge, there is no results about the possibility to simultaneously detect IBP and DCF in the aqueous solutions with an MOF-carbon nanofiber composite electrode. Cyclic voltammetry technique was used for the electrochemical characterization of the electrode in the presence of IBP and DCF. The electroanalytical chronoamperometry and multiple-pulsed amperometry-based protocol for the selective and/or simultaneous detection of IBP and DCF have been developed. 

## 2. Experimental

### 2.1. Electrode Preparation and Characterization

The HKUST metal-organic framework used in the preparation of the composite materials was synthesized by electrochemical methods. 15 mmol (3.15 g) of 1,3,5-benzenetricarboxylic acid (trimesic acid, BTC) and 33 mmol (1.038 g) Tributylmethylammonium methyl sulphate (MTBS) are dissolved in 100 mL of 96%, vol. ethanol (78.5 g). The mixture is heated up to 40 °C in an electrochemical cell with two copper electrodes at a distance of at least 3 cm. A current density of 5 mA/cm^2^ is applied to the electrodes for one hour under continuous stirring. The produced material is filtered off and cleaned with ethanol at room temperature overnight, then filtered again and dried at 100 °C. More details related to the synthesis and morphological characterization have already been published [20].

The HKUST metal-organic framework carbon nanofiber-epoxy composite electrodes were obtained by an effective two-roll mill procedure. The ratios of the two electrodes were chosen to reach 25% wt. carbon nanofiber, 25% wt. HKUST, and 50% wt. epoxy resin.

The procedure for the preparation of HKUST-CNF-epoxy composite electrode is similar to the one applied for the carbon nanofiber-epoxy composite electrode, which was already published elsewhere [8,22].

The morphological characterization was performed using XL 20 Philips Scanning Electron Microscope using an acceleration voltage of 15 KV. The electrical conductivity was determined by four-point probe (FPP) measurements, using a DMM 2000 Digital multimeter and a 6221 DC current source, both provided by Keithley.

### 2.2. Detection Experiments

The electrochemical measurements were carried out with an Autolab potentiostat/galvanostat PGSTAT 302 (Eco Chemie, The Netherlands) controlled with GPES 4.9 software using a three-electrode cell, with a saturated calomel reference electrode, a platinum counter electrode, and HKUST-CNF working electrodes. A HKUST-CNF working disc electrode with 3 mm diameter was mechanically polished and stabilized by repetitive cycling between −0.5 V and +1.25 V vs. SCE in 0.1 M Na_2_SO_4_ supporting electrolyte. The electrochemical behavior of the electrode envisaging IBP and DCF detection was studied by cyclic voltammetry (CV). Chronoamperometry (CA) and multiple-pulsed amperometry (MPA) were used to elaborate the detection protocol for the simultaneous/selective detection of IBP and DCF. 

Stock solution of 0.1 M ibuprofen was prepared from analytical reagent from BASF SE, (Ludwigshafen, Germany) and the stock solution of 0.1 M sodium diclofenac from Amoli Organics Ltd. (Mumbai, India), using distilled water. Sodium sulphate—analytical reagent grade—from Merck was used to prepare 0.1 M Na_2_SO_4_ solution supporting electrolyte. 

## 3. Results and Discussion

### 3.1. Morphostructural and Electrical Characterization

**Figure 1:**
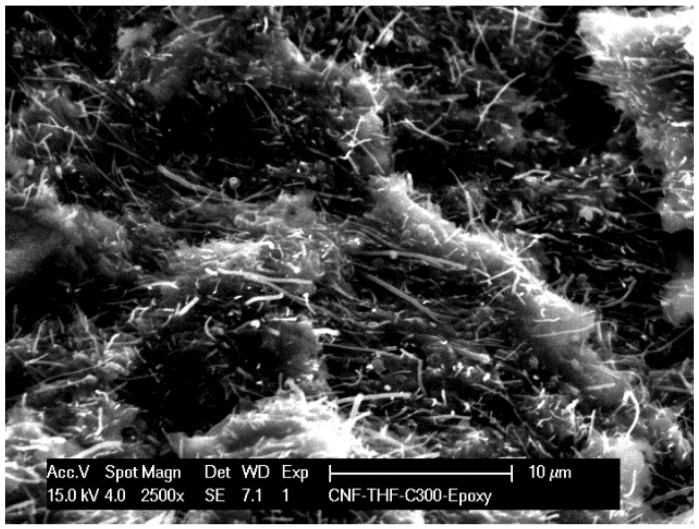
SEM image of the HKUST-CNF composite electrode.

SEM image of the composite surface is shown in Figure 1 and a well and non-homogeneous distribution of both CNF and HKUST within epoxy matrix can be noticed. The electrical conductivity of 0.700 S·cm^−1^ determined by four-point probe resistance measurements revealed its suitability for the electrochemical applications.

### 3.2. Detection Results

Based on our previous results related to the individual detection of DCF and IBP onto the various types of carbon-based composite electrodes [8,10], the aim of this study is to design and to develop specific detection protocols for simultaneous and/or selective detection of DCF and IBP using new MOF(HKUST)-CNF (HKUST-CNF) composite electrodes.

#### Individual Detection of DCF and IBP on HKUST-CNF Electrode

**Figure 2:**
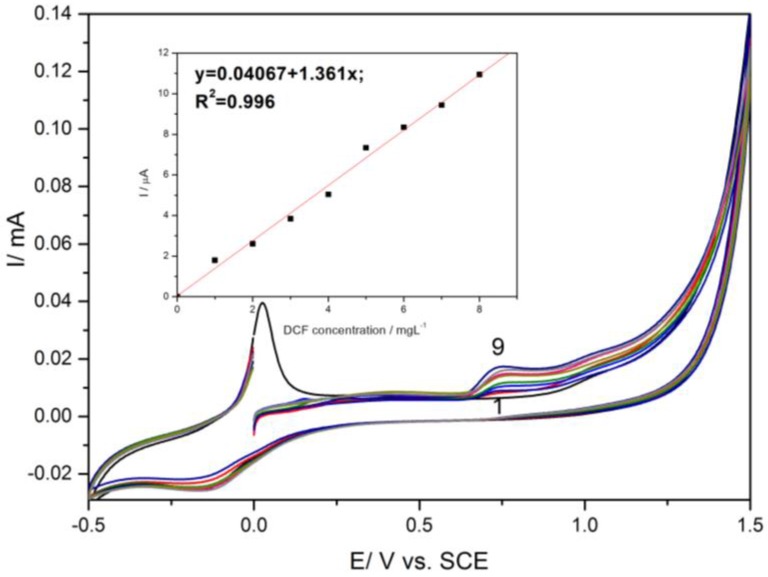
Cyclic voltammogram recorded on HKUST-CNF electrode in 0.1 M Na_2_SO_4_ supporting electrolyte (curve 1) and in the presence of various DCF concentrations: curves 2–9: 1–8 mg·L^−1^ DCF; potential scan rate: 0.05 V·s^−1^; potential range: −0.5 to +1.5 V/SCE. Inset: Calibration plots of the currents recorded at E = +0.75 V vs. SCE vs. DCF concentrations.

**Figure 3:**
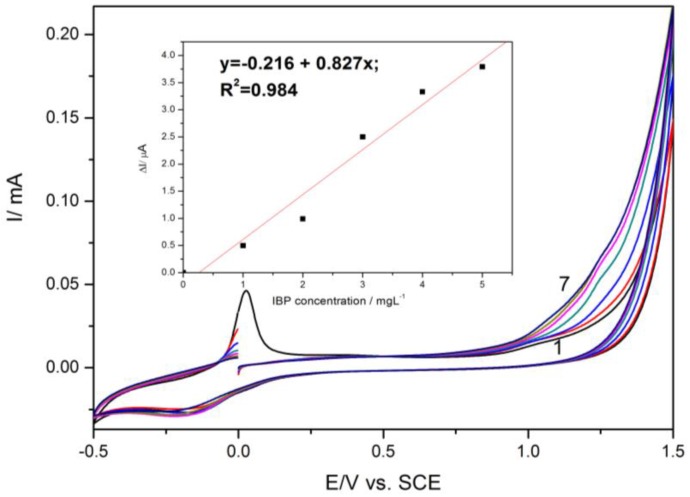
Cyclic voltammogram recorded on HKUST-CNF electrode in 0.1 M Na_2_SO_4_ supporting electrolyte (curve 1) and in the presence of various IBP concentrations: curves 2–7: 1–6 mg·L^−1^ IBP; potential scan rate: 0.05 V·s^−1^; potential range: −0.5 to +1.5 V/SCE. Inset: Calibration plots of the currents recorded at E = +1.25 V vs. SCE versus IBP concentrations.

HKUST-CNF electrode was characterized comparatively by cyclic voltammetry in the presence of various concentrations of DCF (Figure 2) and IBP (Figure 3) within the potential window range, from −0.5 to +1.5 V/SCE. In accordance with the literature [21], CV recorded on HKUST-CNT electrode in supporting electrolyte informed Cu species-based redox processes. The peak, recorded at about 0 V/SCE, corresponding to oxidation process that generates Cu^+^ ions within the lattice of MOF and further oxidation at potential value of about +1.1 V/SCE generating Cu^2+^ ions. On the reverse scanning, the reduction process corresponded to Cu(II) and Cu(I) reduction. The DCF oxidation process occured at the potential value of +0.75 V/SCE and IBP oxidation at the potential value of +1.25 V/SCE. The linear dependences of the anodic peak current were recorded at +0.75 V/SCE versus DCF concentrations (Inset of Figure 2) and at +1.25 V/SCE versus IBP concentrations (inset of Figure 3) were noticed.

The correlation coefficient higher than 0.9 reclaims the utility of this electrode for the detection of each DCF and IBP in the aqueous solutions. Also, the difference between the oxidation peak potentials corresponding to IBP oxidation and a DCF oxidation of about 500 mV shows a great potential for their simultaneous detection, but the question that arises is related to mutual interference.

**Figure 4:**
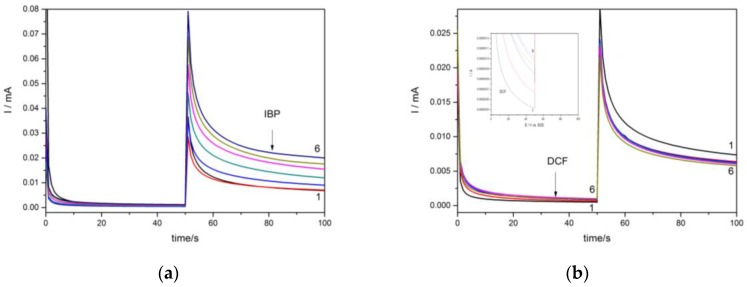
CA recorded under two potential levels of +0.8 V and +1.25 V vs. SCE at the HKUST-CNF electrode in 0.1 M Na_2_SO_4_ supporting electrolyte (curve 1) and in the presence of (**a**) 2 mg·L^−1^ IBP (curve 2), 4 mg·L^−1^ IBP (curve 3), 6 mg·L^−1^ IBP (curve 4), 8 mg·L^−1^ IBP (curve 5), 10 mg·L^−1^ IBP (curve 6); (**b**) 2 mg·L^−1^ DCF (curve 2), 4 mg·L^−1^ DCF (curve 3), 6 mg·L^−1^ DCF (curve 4), 8 mg·L^−1^ DCF (curve 5), 10 mg·L^−1^ DCF (curve 6).

It is well-known that the chronoamperometric technique is the easiest and the most useful for the practical utility, and this technique was tested for the amperometric detection of each IBP and DCF in the aqueous solution. The chronoamperograms were recorded under the operating conditions of two potential levels, one at +0.8 V/SCE and the other at +1.25 V/SCE corresponding to DCF and respectively, IBP detection, in accordance with CV results considered the reference basis for the amperometric detection set-up. The two-potential levels for operating chronoamprograms were recorded for the individual detection of each pharmaceutical in order to check the potential interference effect of one pharmaceutical to the other’s detection. The results are presented in Figure 4, and it can be noticed that there is no contribution from one pharmaceutical to the signal corresponding to the other pharmaceutical. However, a slight decrease of the current recorded at +1.25 V/SCE in the presence of DCF is noticed, probably due to the effect of the Cu(II) generation process.

For each pharmaceutical, there is a linear dependence between the current recorded at a certain potential value and pharmaceutical concentrations and the sensitivities are gathered in Table 1. It can be seen that, by the chronoamperometry, the sensitivity decreased for DCF and increased for IBP. An explanation for this phenomenon is a possible carbon electrode fouling at the potential value of +0.75 V/SCE in comparison with the higher potential (+1.25 V/SCE) demanded for IBP oxidation and detection. The higher potential value assurred a cleaning of the electrode surface by the advanced oxidation.

In order to improve the electroanalytical characteristics for the detection of each pharmaceutical, multiple-pulsed amperometry (MPA) was applied under the optimization conditions established for IBP. The four-potential levels were applied for MPA in accordance with the CV shape:

−0.2 V for the time duration of 0.5 s, which assumes assurance of electrode preconditioning;
+0.8 V for the time duration of 0.5 s, which is characterstic of DCF oxidation;
+1.25 V for the time duration of 0.1 s, which is characteristic of IBP detection;
+0.02 V for 0.5 s, corresponding to copper oxidation.

**Figure 5:**
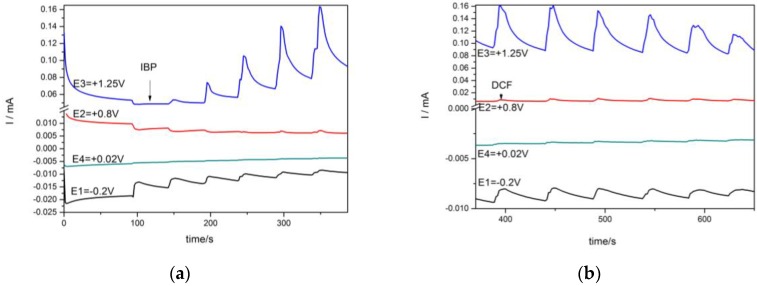
Multiple-pulsed amperograms recorded by HKUST-CNF electrode in 0.1 M Na_2_SO_4_ supporting electrolyte and adding consecutively and continuously: (**a**) 2 mg·L^−1^ IBP, recorded at E1 = −0.2 V/SCE, E2 = +0.8 V/SCE, E3 = +1.25 V/SCE, E4 = +0.02 V/SCE; (**b**) 2 mg·L^−1^ DCF, recorded at E1 = −0.2 V/SCE, E2 = +0.8 V/SCE, E3 = +1.25 V/SCE, E4 = +0.02 V/SCE.

The amperograms are presented in Figure 5, and the sensitivities are gathered in Table 1. 

**Figure 6:**
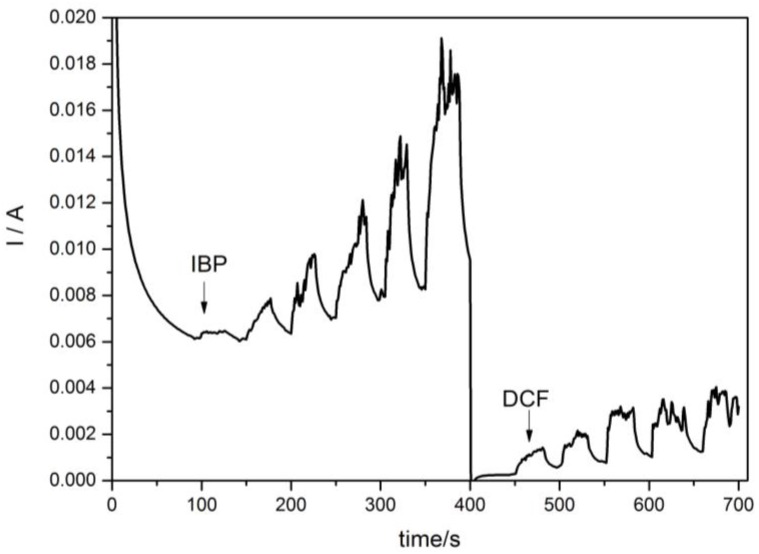
Amperometric response at the HKUST-CNF electrode for the six successive and continuous additions of 2 mg·L^−1^ IBP recorded at +1.25 V vs. SCE for 400 s running time followed by the five successive and continuous additions of 2 mg·L^−1^ DCF at applied potential of +0.8 V vs. SCE for the next running time of 400 s.

The results of the individual detection of each IBP and DCF are very promising for their simultaneous or selective detection in relation to the detection potential value. The detection separation higher than 250 mV should make the simultaneous detection possible. Taking into account the higher sensitivity for IBP detection and higher value of the detection potential, the question that arises is whether or not the DCF can be detected in the presence of IBP. Using a very simple detection scheme using chronoamperometry by continuous and successive addition of IBP in the first step recorded at +1.25 V/SCE followed by the second step recorded at the +0.8 V/SCE for DCF, it was proven that DCF should be detected in the presence of IBP by easily switching the detection potential value (Figure 6). 

This detection scheme allowed better sensitivity for determining DCF detection (0.186 µA/mgL^−1^) versus the sensitivity for determining the individual DCF with CA (0.050 µA/mgL^−1^). This aspect should be explained by the enhanced hydrodynamic conditions assured by continuous stirring conditions and by the quasi-preconditioning/cleaning conditions, which were applied for IBP detection, and which affected the electrode surface cleaning. This detection scheme is very useful as selective detection of each pharmaceutical in the presence of another by the detection of potential selection.

MPA technique can be explored to elaborate various detection schemes based on the potential level numbers, values, and operating time, either for simultaneous or selective detection of the pharmaceuticals, characterized by enhanced sensitivities. Four-level-based. multiple-pulsed amperometry was suitable to be able to detect each IBP and DCF at the same time. For two-level, and three-level-based multiple-pulsed amperometry, no signal for both pharmaceuticals was found (the results are not shown here).

For four-level-based MPA, the operating time was the control parameter for simultaneous/selective detection and also influenced the electroanalytical performance of the detection. This time represents in fact, the oxidation time allocated to each oxidation process for IBP and DCF. 

**Figure 7:**
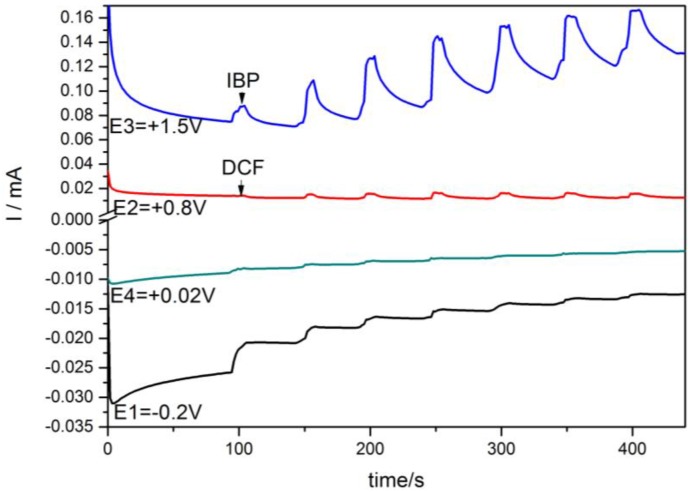
Multiple-pulsed amperograms recorded with a HKUST-CNF electrode in 0.1 M Na_2_SO_4_ supporting electrolyte and continuously adding a mixture of 2 mg·L^−1^ DCF and 2 mg·L^−1^ IBP, recorded at E1 = −0.2 V/SCE (time duration of 0.5 s), E2 = +0.8 V/SCE (time duration of 0.5 s), E3 = +1.25 V/SCE (time duration of 0.1 s), E4 = +0.02 V/SCE (time duration of 0.5 s).

For simultaneous detection of IBP and DCF, maintaining the short oxidation time for DCF led to enhancing the DCF sensitivity under longer oxidation time for IBP (see Table 2). This should be explained by avoiding the electrode fouling during DCF oxidation due to IBP oxidation that occurred at the higher oxidation potential in the presence of HKUST-1 catalyst, also involving the O_2_ evolution starting, which allowed electrode surface cleaning. To improve IBP detection, the time duration for IBP oxidation was shortened and the time duration dedicated to DCF was longer. These conditions are the same to those previously presented for the individual detection of IBP and DCF, and the results are presented in Figure 7. The sensitivities are shown in Table 2. To check the reproducibility of the results, the same detection protocol was applied under the conditions of consecutively adding the pharmaceuticals and the same sensitivities were found, which confirmed the reproducibility (the results are not shown here).

Various detection protocols based on the four-potential levels have been tested for the selective detection of IBP and DCF. The better sensitivity represents the second criteria for setting-up the operating conditions for multiple-pulsed amperometry. To assure the selective detection, the most positive potential value was modified, +1.5 V/SCE was used instead of 1.25 V/SCE. Also, the potential level position influenced the detection characteristics. For the selective detection of IBP, the detection protocol consisted of:

−0.20 V for 0.5 s, which assures electrode preconditioning ;
+1.50 V for 0.2 s, which is characterstic to DCF oxidation;
+0.80 V for 0.2 s, which is characteristic to IBP detection;
+0.02 V for 0.5 s, corresponding to copper oxidation.

**Figure 8:**
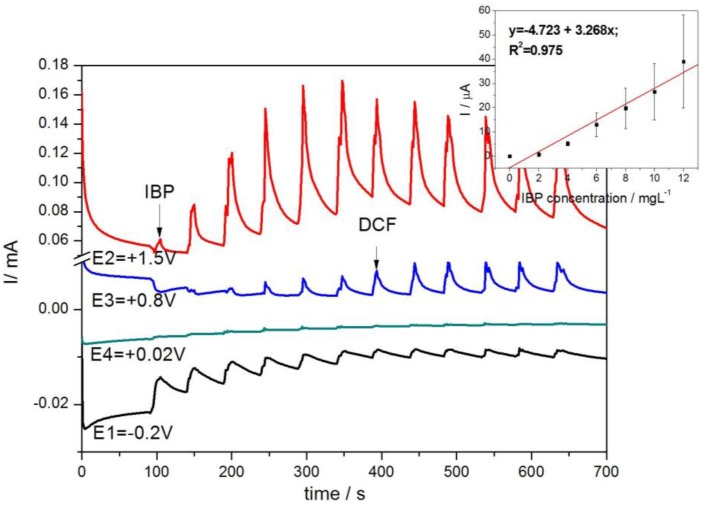
Multiple-pulsed amperograms recorded at HKUST-CNF electrode in 0.1 M Na_2_SO_4_ supporting electrolyte, consecutively and continuously adding each of 2 mg·L^−1^ IBP and respective, DCF, recorded at E1 = −0.2 V/SCE, E2 = +1.5 V/SCE, E3 = +0.8 V/SCE, E4 = +0.02 V/SCE. Inset: Calibration plots of the currents recorded at E = +1.5 V vs. SCE versus IBP concentrations.

The multiple-pulsed amperograms are presented in Figure 8, and it can be noticed that the selective detection of IBP is achieved at a potential value of +1.5 V/SCE, and the corresponding calibration plots is shown in inset. 

The selective detection of DCF was achieved under operating conditions of:

−0.20 V for 0.5 s, which assures electrode preconditioning;
+1.50 V for 0.1 s, which is characterstic to DCF oxidation;
+0.80 V for 0.1 s, which is characteristic to IBP detection;
+0.02 V for 0.5 s, corresponding to copper oxidation.

**Figure 9:**
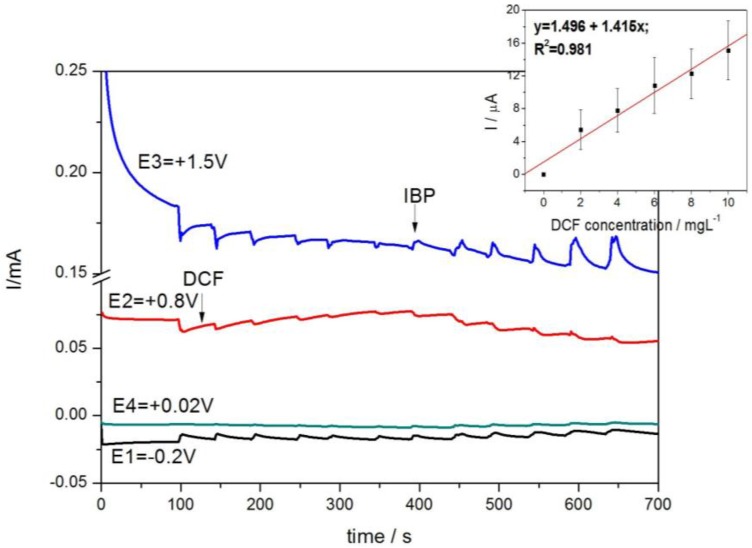
Multiple-pulsed amperograms recorded at HKUST-CNF electrode in 0.1 M Na_2_SO_4_ supporting electrolyte and consecutively and continuously adding each of 2 mg·L^−1^ DCF and respective, IBP, recorded at E1 = −0.2 V/SCE, E2 = +0.8 V/SCE, E3 = +1.5 V/SCE, E4 = +0.02 V/SCE. Inset: Calibration plots of the currents recorded at E = +0.8 V vs. SCE versus DCF concentrations.

In Figure 9 we show multiple-pulsed amperograms, and it can be noticed that under these operating conditions only DCF was detected. The linear dependence of the signal versus DCF concentrations is presented in inset. 

It should be noticed that the potential value, pulse time, and level number and order represent very important working parameters for the development of the detection strategy. 

All electroanalytical parameters determined for all developed detection protocols regarding the selective/simultaneous detection are gathered in Table 2. Similar sensitivities for the simultaneous/selective detection of IBP and DCF, respectively, were achieved for MPA technique. MPA improved the electroanalytical parameters for the IBP and DCF detection.

The stability of the electrode was also investigated by CV at the time intervals of 10, 20, and 30 days and the peak currents were modified by 1.5% for DCF and 2.1% for IBP, indicating a high stability of the electrode. The reproducibility was tested by MPA for three replicates and RSD of 2.5% for IBP and 2.6% for DCF showed a good reproducibility. The recovery studies were carried out by spiking the water with a known amount of DCF and IBP (2 mg·L^−1^) using MPA-based protocol optimized for the simultaneous detection. The analysis of DCF and IBP in water exhibited a mean recovery of 99.5% for DCF and 99.0% IBP, and a relative standard deviation of 2.8% for IBP and 3.5% for DCF—indicating adequate precision and accuracy of this electrochemical method.

## 4. Conclusions

The composite electrode of HKUST-1 metal-organic framework-carbon nanofiber within an epoxy matrix (HKUST-CNF) has been prepared in this study. A well and non-homogeneous distribution of both CNF and HKUST within an epoxy matrix was noticed by SEM imaging, and its electrical conductivity of 0.700 S·cm^−1^ revealed its suitability for the electrochemical application. This electrode was tested for the individual, selective, and/or simultaneous detection of ibuprofen (IBP) and diclofenac (DCF). The voltammetric parameters related to the oxidation potential and, as a consequence, the detection potentials were determined by cyclic voltammetry technique, which was considered as a reference basis for developing the detection strategy based on the amperometric techniques. Individual detection of IBP and DCF was achieved through CV, CA, and MPA. For the individual detection of IBP, the best electroanalytical parameters expressed in terms of sensitivity and the lowest limits of detection were achieved by amperometric techniques (CA and MPA). CV attained the best sensitivity for the individual detection of DCF and the lowest limit of detection using MPA. This electrode allowed selective detection of IBP and DCF by simply switching the detection potential. However, the MPA operated under optimum working conditions of four-potential levels selected, based on CV shape, and allowed a significant enhancement of the sensitivity. Moreover, MPA operated within four potential level based detection protocol under optimum operating conditions related to the potential value, pulse time, and level number and order led to the simultaneous detection of IBP and DCF characterized by the enhanced detection performance.

## Figures and Tables

**Table 1:** The electroanalytical parameters reached for the individual detection of IBP and DCF with HKUST-CNF electrode.

Technique	Compound	Conditions/E, V/SCE	Sensitivity µA/mg·L^−1^	Correlation Coefficient (R^2^)	LOD ^a^ (µg·L^−1^)	LQ ^b^ (µg·L^−1^)	RSD ^c^ (%)
CV	IBP	+1.25 V	0.827	0.984	21.70	72.00	2.05
	DCF	+0.74 V	1.361	0.996	100	333	0.70
CA	IBP	+1.25 V	1.201	0.987	0.13	0.46	0.81
	DCF	+0.8 V	0.050	0.954	1.59	5.29	0.52
MPA	IBP	−0.2 V—0.5 s +0.8 V—0.5 s +1.25 V—0.1 s +0.02 V—0.5 s	4.541	0.969	4.0	1.3	1.14
	DCF	−0.2 V—0.5 s +0.8 V—0.5 s +1.25 V—0.1 s +0.02 V—0.5 s	0.103	0.982	2.08	6.96	1.19

^a^ The lowest limit of detection determined in according with the literature [22]; ^b^ The lowest limit of quantification determined in according with the literature [22]; ^c^ For three replicates.

**Table 2:** The electroanalytical parameters reached for the selective/simultaneous detection of IBP and DCF with a HKUST-CNF electrode.

Detection	Compound	Technique/Conditions	Sensitivity µA/mgL^−1^	Correlation Coefficient (R^2^)	LOD (µg·L^−1^)	LQ (µg·L^−1^)	RSD (%)
Simultaneously	IBP	CA/E = +1.25 V	1.107	0.963	8.01	27.10	0.05
		MPA/ E1 = −0.2 V—0.5 s; E2 = +0.8 V—0.5 s; E3 = +1.25 V—0.1 s; E4 = +0.02 V—0.5 s	5.166	0.990	3.20	1.10	1.05
	DCF	CA/E = +0.8 V	0.186	0.991	49.2	164	1.24
		MPA/ E1= −0.2 V—0.5 s; E2 = +0.8 V—0.5 s; E3 = +1.25 V—0.1 s; E4 = +0.02 V—0.5 s	0.095	0.988	11.9	39.8	2.69
Selective	IBP	MPA/ E1 = −0.2 V—0.5 s; E2 = +1.5 V—0.2 s; E3 = +0.8 V—0.2 s; E4 = +0.02 V—0.5 s	3.268	0.975	6.10	2.00	1.17
	DCF	MPA/ E1 = −0.2 V—0.5 s; E2 = +0.8 V—0.1 s; E3 = +1.5 V—0.1 s; E4 = +0.02 V—0.5 s	1.415	0.981	3.20	10	2.11
